# ’Malignant’ hypertension from hyperaldosteronism: a case report

**DOI:** 10.11604/pamj.2018.30.10.14015

**Published:** 2018-05-04

**Authors:** Krishna Mohan Baradhi, Thao Tran, Penchala Swamy Mittadodla

**Affiliations:** 1 University of Oklahoma, Department of Internal Medicine/Nephrology, Tulsa Schusterman Center, Tulsa, Oklahoma, USA, 74135; 2 Mercy Clinic, Critical Care Medicine, Rogers, Arkansas, USA, 72758

**Keywords:** Secondary hypertension, hyperaldosteronism, adrenocortical carcinoma, hypokalemia

## Abstract

Adrenocortical carcinomas (ACC) are rare with an incidence of 0.7-2 per million population per year and account for only 0.05%-2% of all malignant tumors. While majority of the functional ACC present as Cushing syndrome, recurrent hyperaldosteronism from metastatic ACC is exceedingly rare. We describe a 67-year old female presented with hypertensive urgency & hypokalemia as a result of hyperaldosteronism from an 8-cm right ACC. She underwent a radical right nephrectomy with adrenalectomy that normalized her blood pressure. However, a few years later she presented again with resistant hypertension from hyperaldosteronism, raising the suspicion of recurrence of ACC. A contrast-enhanced CT scan showed a normal left adrenal gland but revealed pulmonary metastases of ACC based on a lung biopsy. Chemotherapy was complicated with side effects leading to refusal of further chemotherapy, henceforth requiring high dose of spironolactone for blood pressure control. Despite curative surgery, metastatic functional ACC should be considered in patients presenting with secondary hypertension from recurrent hyperaldosteronism, due to its high recurrence rate. Besides standard cancer surveillance after a curative surgery, meticulous monitoring of blood pressure is a simple yet crucial way to detect cancer recurrence early.

## Introduction

Adrenocortical carcinomas (ACC) are rare malignancy with an incidence of 0.7-2 per million population per year [1]. While ~60% of ACC are functional, clinical manifestations of hyper-secretion are seen in only ~40% of the cases. Hormone-secreting ACC usually present as Cushing´s syndrome (45%), or a mixed Cushing's and virilization syndrome, (25%), whereas, aldosterone-producing tumors are exceptionally rare comprising only ~2.5% of ACC. Amongst these unfamiliar territories, hyperaldosteronism from metastatic adrenal carcinoma has rarely been reported. We herein present a first unique case of secondary hypertension as a result of hyperaldosteronism arising from lung metastasis of the previously resected ACC.

## Patient and observation

**Figure 1:**
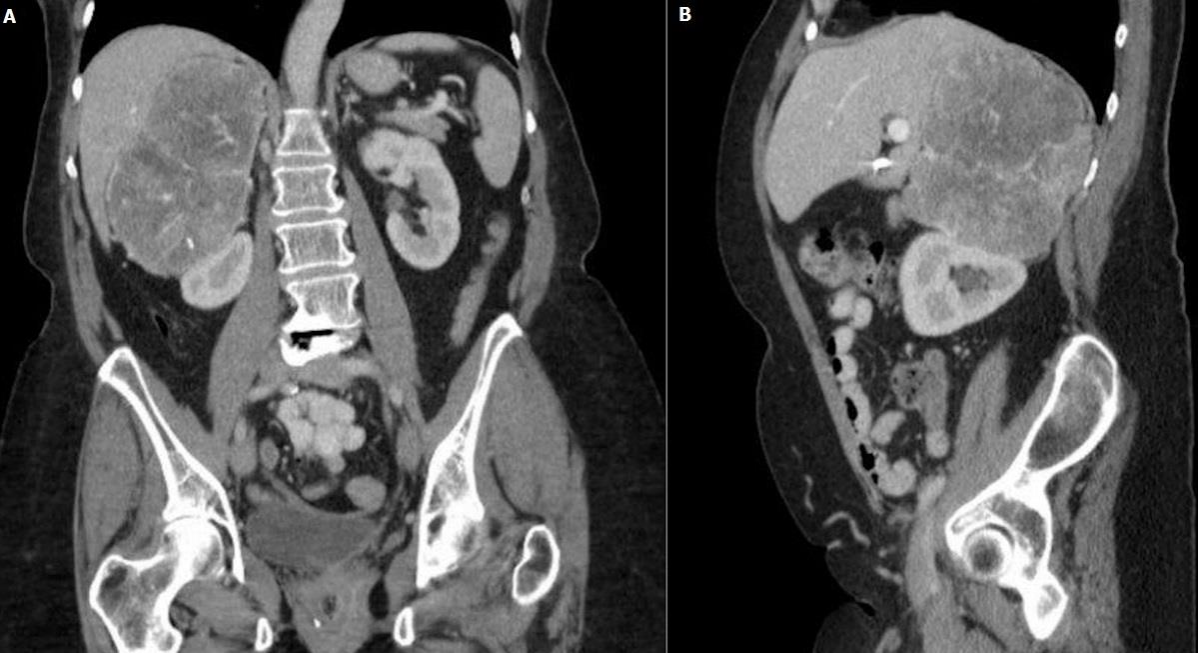
CT abdomen: (A) coronal section showing 8cm mass above the right kidney; (B) sagittal section showing the adrenal tumor

**Figure 2:**
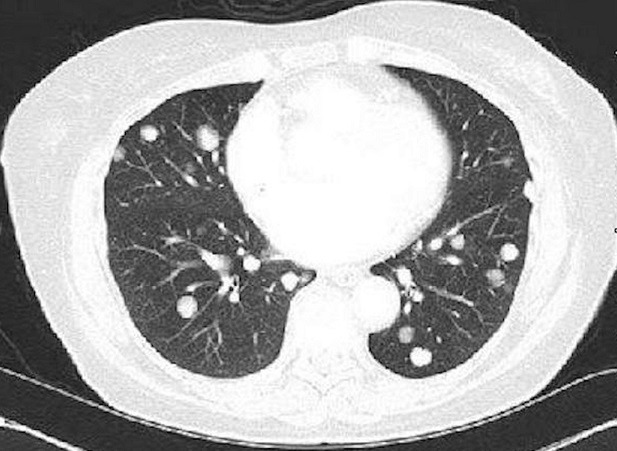
CT chest showing bilateral pulmonary metastases

The patient is a 67-year-old Caucasian woman who presented with uncontrolled hypertension. Patient did not have major medical issues until her early 60s when she started noticing rise in blood pressure during her routine clinic visits. Physical examination was within the normal limits except her systolic blood pressure was consistently in the 200s. Laboratory evaluation was significant for hypokalemia of 2.7 meq/L, bicarbonate -24 meq/L and creatinine of 0.92 mg/dl. Further evaluation for possible etiology of secondary hypertension revealed high aldosterone-renin ratio. Computed tomography (CT) scan to evaluate hyperaldosteronism showed a large mass in right adrenal gland measuring 10 x 7.8 cm (Figure 1). Further FDG-PET scan showed a right adrenal mass and periceliac lymph nodes were positive. The patient underwent a radical right adrenalectomy with nephrectomy and lymph nodes dissection. Pathology revealed 14cm adrenal cortical carcinoma with two lymph nodes next to vena cava which were positive. She also completed adjuvant radiation and chemotherapy. Her blood pressure and serum potassium normalized after adrenalectomy. Follow up FDG-PET scan a year later, showed increase glucometabolism in a nodal mass of the left paratracheal region superior to the clavicle and underwent lymph node dissection. Biopsy again showed metastatic ACC of supraclavicular lymph node. Patient completed 3 cycles of carboplatin and etoposide. In addition, during this time, the patient also diagnosed with papillary thyroid carcinoma for which she underwent thyroidectomy and ablation. Blood pressure was within the normal limits during this period. Patient was lost to follow up for two years due to non-compliance until she presented back again with difficult to control blood pressure with associated hypokalemia similar to her prior presentation (Table 1). Her medications at this time included clonidine, metoprolol, Lisinopril, Torsemide, and potassium supplements. Despite these regimen, her systolic blood pressure remained in the 200s. Further testing revealed aldosterone-renin ratio of greater than 200, raising the red flag of possible recurrence of ACC. A CT abdomen showed normal left adrenal gland, however CT chest revealed multiple pulmonary lesions favoring metastasis as shown in the Figure 2. Initial bronchoscopy showed many atypical cells but no malignancy. Percutaneous lung biopsy unveiled these lesions to be of metastatic cancer of adrenocortical origin. She underwent chemotherapy again complicated with side effects leading to refusal of further chemotherapy, henceforth requiring high dose of spironolactone (400 mg daily) for blood pressure control.

**Table 1:** Laboratory data

Chemistry	Initial presentation	Recurrent presentation	Units
Sodium	136	140	mEq/L
Potassium	2.7	3.1	mEq/L
Chloride	104	106	mEq/L
Bicarbonate	24	25	mEq/L
Calcium	8.7	8.8	mEq/L
Creatinine	0.92	1.13	mEq/L
BUN	14	15	mg/dL
Glucose	133	88	mg/dL

Aldosterone/Renin ratio >200

## Discussion

Secondary hypertension from aldosterone secreting ACC is not only uncommon but may also be unfamiliar to the practicing clinicians. Amongst these odd etiologies of hyperaldosteronism, pulmonary metastasis of previously resected ACC is a rare entity. Deckers et al reported a case of hyperaldosteronism from peritoneal dissemination of the previously removed ACC [2]. Calvo-Romero et al reported a case of resistant hypertension as a result of hyperaldosteronism from recurrent adrenal adenoma [3]. ACC usually affect middle aged group and are slightly more common in females [4]. The clinical presentation of aldosterone producing ACC is hypertension and hypokalemia. It is clinically indistinguishable from the Conn's syndrome presentation and pathological differentiation can be challenging. Both size and appearance on imaging are used to distinguish between benign and malignant adrenal mass. Tumors > 6cm are highly suspicious for malignancy and require surgical resection according to NIH [5]. For tumors between 4 and 6 cm, additional criteria should be considered before adjudicating to monitor or proceed to adrenalectomy [5]. In addition, Weiss criteria can also be used to distinguish between benign and malignant ACC. Weiss system mandates meeting three out of the nine histological features to be regarded as malignant [6]. Amongst them, the presence of atypical mitoses, capsular invasion, tumor weight greater than 250g, and size greater than 10cm each showed a statistical association with poor survival, compared to other features, such as nuclear grade, presence of necrosis or of venous /sinusoidal invasion, character of the tumor cell cytoplasm, or architectural pattern, showed no statistical significance in predicting survival [7].

Mineralocorticoid antagonists or adrenalectomy can be the option of treatment for classical causes of primary hyperaldosteronism, whereas the only potential lifesaving option for ACC is complete radical resection [8]. It is imperative that hormonal assessment is done prior to adrenalectomy. Despite surgical resection, there is a high recurrence rate of ACC, up to 74% recurrence rate either local or distant metastasis [9]. Hence, Fassnacht et al advocate periodic radiological and biochemical monitoring for at least 10 years post-surgery for ACC [4]. For aldosterone producing ACC, common sites of metastasis include liver, lung, abdominal lymph modes, contralateral adrenal and abdominal lymph nodes [10]. The prognosis of ACC depends on tumour stage and is generally poor. Interval between the initial resection of an adrenocortical carcinoma and recurrence can dictate prognosis [11]. The overall 5-year survival rate range between 16 and 38% and median survival for metastatic disease is less than 12 months [12,13]. Largest study to date indicated rates of response and progression-free survival were significantly better with etoposide plus mitotane than with streptozocin plus mitotane as first-line therapy for advanced ACC [14]. Our patient presented with recurrent metastatic ACC with resultant hyperaldosteronism leading to resistant hypertension and hypokalemia. Given the extent of her disease with multiple recurrences of metastasis, mitotane chemotherapy was offered to patient along with the etoposide. Unfortunately, as she succumbed to the side effects of therapy, patient decided against further treatment and required escalating doses of spironolactone to control her blood pressure.

## Conclusion

Our case highlights the importance of considering functional ACC in patients presenting with hyperaldosteronism. Clinicians should be cognizant of high rate of recurrence of ACC and incorporate blood pressure monitoring besides standard radiographic and laboratory surveillance after a curative surgery to detect recurrence of ACC early.

## Competing interests

The authors declare no competing interests.
